# A Coach-Supported mHealth Lifestyle Intervention to Reduce Dementia Risk in Persons With Low Socioeconomic Status or a Migration Background: Qualitative Co-Design Study

**DOI:** 10.2196/76094

**Published:** 2025-11-04

**Authors:** Anne Roos van der Endt, Josephine E Lindhout, Joshua van Apeldoorn, Richler Amponsah, Rayn Ramkishun, Edanur Sert, Casper Craamer, Edo Richard, Marieke P Hoevenaar-Blom, Eric P Moll van Charante

**Affiliations:** 1 Department of Public and Occupational Health Amsterdam University Medical Centers Amsterdam The Netherlands; 2 Department of Neurology Donders Institute for Brain, Cognition and Behaviour Radboud University Medical Center Nijmegen The Netherlands; 3 Department of General Practice Amsterdam University Medical Centers Amsterdam The Netherlands; 4 IQ Health Science Department Radboud University Medical Center Nijmegen The Netherlands; 5 Interactive Studios 's-Hertogenbosch The Netherlands

**Keywords:** mHealth intervention, lifestyle, dementia, low-SES, migration background, tailoring, socioeconomic status

## Abstract

**Background:**

The prevalence and incidence of dementia are higher in migrants and those with low socioeconomic status (SES). Mobile health (mHealth) interventions offer a potentially scalable way to reduce dementia risk via risk factor modification.

**Objective:**

We co-designed the MIND-PRO app—an mHealth intervention targeting dementia risk factors through self-managed lifestyle changes and remote coaching—specifically designed for Dutch individuals with low SES and those with Turkish or South Asian Surinamese migration backgrounds. We focused on these migrant populations as they are the largest in the Netherlands and have the highest risk of developing dementia.

**Methods:**

In this qualitative study, we explored the needs and preferences of our target populations aged 50-75 years old at increased dementia risk by conducting semistructured interviews and focus groups. Participant feedback was used to iteratively refine and adapt a prototype intervention based on insights from prior mHealth trials.

**Results:**

We interviewed 23 participants (median age 59, IQR 55-63 y; n=15, 65% female) and conducted two focus groups with 7 Turkish women and 13 Dutch participants with low SES. The target populations emphasized personalization features such as goal setting, self-tracking, educational material, and remote coaching. Participants highlighted the importance of social interaction and autonomy in achieving sustainable lifestyle changes. Tailoring coaching and lifestyle advice to cultural practices was deemed beneficial.

**Conclusions:**

Optimal mHealth interventions targeting dementia risk factors in migrants and individuals with low SES should be personalized and interactive, respect autonomy, and integrate cultural needs and preferences.

## Introduction

The global prevalence of dementia is expected to increase due to population aging and growth, with up to 40% of cases linked to modifiable risk factors such as high blood pressure, physical inactivity, unhealthy diet, overweight, and smoking [1]. High-risk populations, including individuals of lower socioeconomic status (SES) and those with a migration background in high-income countries, are particularly vulnerable due to clustering of lifestyle-related risk factors, which provides a significant opportunity for prevention [2-7].

Mobile health (mHealth), which involves using mobile communication devices (eg, smartphones, tablets, and smartwatches), may be an effective medium for interventions as it can reach many people in a cost-effective way [8]. In the Healthy Aging through Internet Counseling of the Elderly (HATICE) [9] and Prevention of Dementia by Mobile Phone Applications (PRODEMOS) trials [10], our research team focused on self-management of lifestyle-related cardiovascular and dementia risk factors through interventions delivered via computer, tablet, or smartphone. Key findings from these studies formed the basis for the current prototype of the intervention, the MIND-PRO app. This new intervention is specifically designed to target individuals at increased risk of dementia, particularly those with lower SES or a Turkish or South Asian Surinamese background in The Netherlands. Individuals with a Turkish or South Asian Surinamese background have the highest age-specific prevalence of dementia among groups with a migration background as compared to the majority population, and represent large migrant groups in the Netherlands [4]. The needs and preferences for an mHealth intervention may vary between these groups.

mHealth users consistently highlight the importance of personalized content, user friendliness, human guidance, and autonomy in mHealth interventions [11,12]. Involving end users in the development process can address these needs and enhance engagement and effectiveness [13-15]. Therefore, we used a qualitative co-design study to investigate how a blended mHealth lifestyle intervention can be tailored to effectively reach and engage individuals with lower SES or a Turkish or South Asian Surinamese background.

## Methods

### Study Design

**Figure 1:**
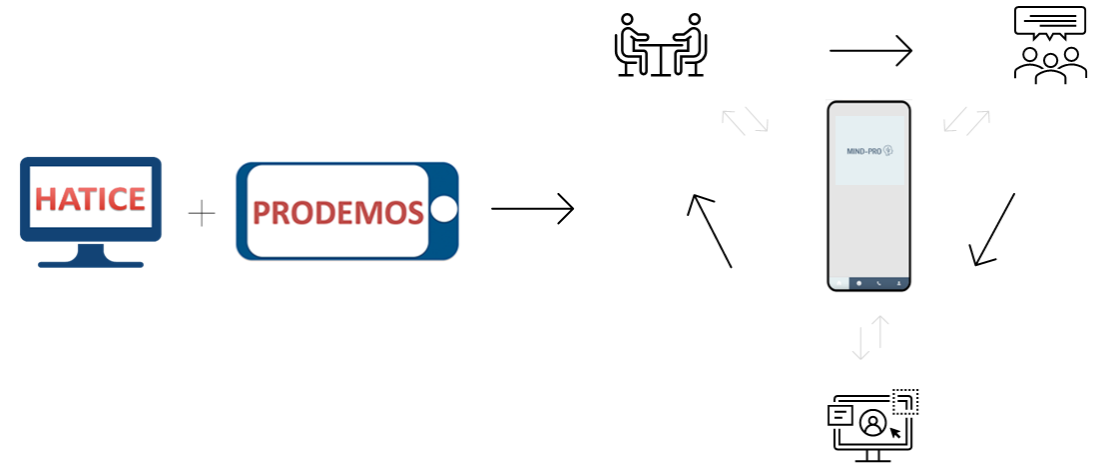
A prototype of the intervention was designed, in collaboration with the mHealth developers and the research team, based on prior eHealth and mHealth trials. This prototype was then presented to participants during interviews and focus groups, after which their feedback was used in iterative cycles to make adjustments to the prototype. This process ultimately led to the final version of the intervention. HATICE: Healthy Aging through Internet Counseling of the Elderly; mHealth: mobile health; PRODEMOS: Prevention of Dementia by Mobile Phone Applications.

This qualitative study was based on a prototype of the blended mHealth lifestyle intervention—The MIND-PRO app—built on insights from the HATICE and PRODEMOS trials [9,16-21]. To ensure usability and cultural relevance, we actively involved our target population by asking for their feedback on the prototype. In iterative cycles of feedback, the researchers and mHealth developers refined the intervention according to participants’ input. This co-design process incorporated culture-specific needs and preferences, aiming to align the intervention with its target population (Figure 1).

Initially, semistructured interviews were conducted to explore participants’ perspectives and requirements for a coach-supported lifestyle intervention. Among Turkish participants, we observed diverse views on culturally sensitive approaches. To gain a deeper understanding of these different perspectives, we organized a focus group to facilitate further discussion and refinement of the platform, ensuring that it was both user-friendly and culturally appropriate. The perspectives of Dutch citizens with low SES perspectives on mHealth interventions had already been qualitatively explored in a previous study [20] and tested in a randomized controlled trial for efficacy and implementation [16]. Therefore, in this study, we directly tested the MIND-PRO prototype in a focus group with this population. This study adhered to the COREQ (Consolidated Criteria for Reporting Qualitative research) checklist (Multimedia Appendix 1).

### Study Population and Recruitment

Participants were community-dwelling Dutch individuals with low SES or a Turkish or South Asian Surinamese background. We used convenience sampling by recruiting participants from community centers, with the help of key figures and the authors’ networks. For the Dutch or nonmigrant group, low SES was defined based on self-reported educational attainment, categorized as none, only primary education, or lower vocational or lower secondary education. A Turkish or South Asian Surinamese background was defined as having at least one parent born in Turkey or Suriname. Inclusion criteria were being 50-75 years old, owning a smartphone, and having at least one dementia risk factor (hypertension, dyslipidemia, diabetes mellitus, physical inactivity, smoking, depression, or being overweight). We acknowledge that referring to “individuals with Turkish or South Asian Surinamese backgrounds” may insufficiently represent the diversity within the group. However, specifying cultural background is essential to tailor the intervention effectively [22]. Similarly, using SES as a criterion may not fully capture individuals’ competencies and may be perceived as stigmatizing [23]. Our rationale for these definitions is that these groups are underrepresented in research, often leading to scarce and ineffective interventions targeting their specific needs.

### Prototype of the mHealth Intervention

**Figure 2:**
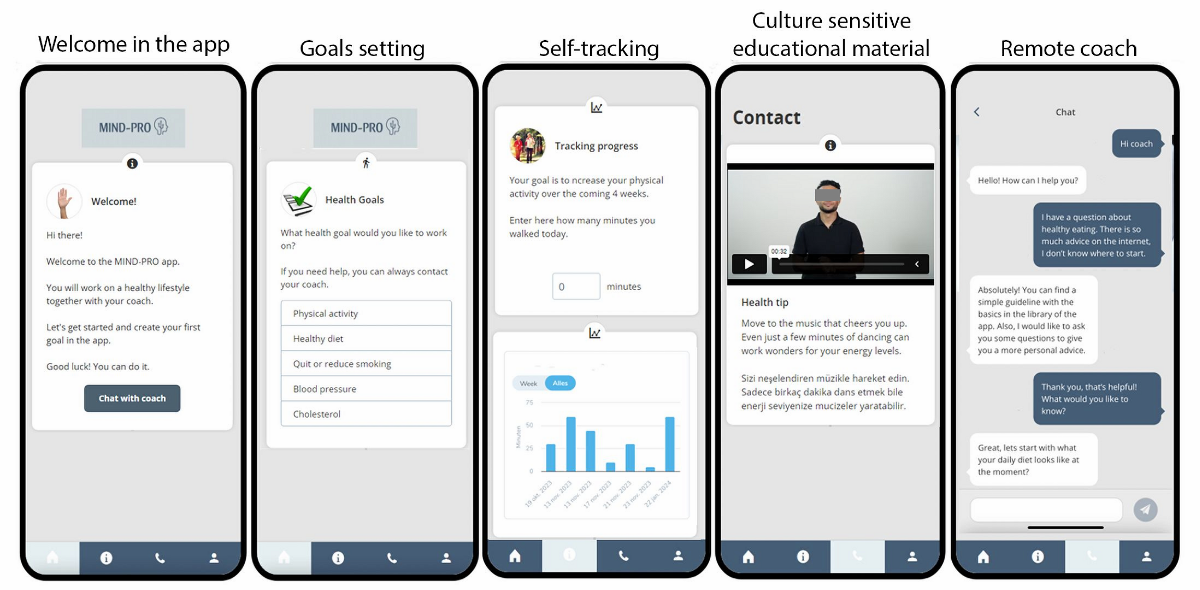
Overview of some key functionalities available in the app. Users can set lifestyle goals, track their progress, receive videos from their personal coach, and chat with their coach.

The intervention design was guided by Bandura’s Social Cognitive Theory, which informed the goal-setting and self-tracking structure [24]. To enhance perceived ease-of-use and usefulness, we applied the frameworks of the Technology Acceptance Model [25] and Unified Theory of Acceptance and Use in Technology [25]. In the prototype, participants could set lifestyle change goals, record self-measurements, and monitor their progress, for example, on bodyweight, blood pressure, and physical activity duration and frequency. Progress was visually represented through graphs and color-coded diaries. Additionally, participants had the option to send messages to a coach, and the app featured a library of education materials specifically designed for this study (Figure 2).

### Data Collection

Interviews and focus groups were conducted by six members of the research team: ARE (Dutch, female, MD), JEL (Dutch, female, MD), RR (Dutch, male, anthropologist, South Asian Surinamese migration background), RA (Dutch, female, master’s student, Ghanaian migration background), ES (Dutch, female, research coordinator, Turkish migration background), and MPH-B (Dutch, female, postdoctoral researcher). We worked with a multicultural team to promote alignment between interviewers and participants in terms of language and cultural background. Interviewing experience ranged from novice to highly experienced. Interviews and focus groups were conducted in Dutch or Turkish, with ES providing translation when necessary [26].

Topic lists (Multimedia Appendices 2 and 3) were developed by ARE and JL under the supervision of senior qualitative researchers (EPMC and MPH-B), drawing on the Technology Acceptance Model and Unified Theory of Acceptance and Use in Technology models [25]. ES contributed to ensuring accurate translation and clarification of concepts.

Interviews and focus groups began with general discussions on lifestyle perceptions, past behavior changes, and experiences with health apps. The intervention prototype was then introduced with minimal guidance, allowing participants to explore it independently or in small groups while researchers observed usability. Open-ended questions explored key features: user-friendliness, goal setting, progress tracking, coaching, and educational materials. Participants provided input on necessary adjustments to better fit their needs and preferences. Identified themes related to culture and gender were tested across groups. The interview guide was iteratively refined throughout data collection (Multimedia Appendices 2 and 3).

Adjustments to the app were made in real time, allowing participants to immediately confirm whether their preferences were understood. Most interviews were conducted individually, though four small-group interviews (up to four participants) were held at participants’ request, primarily due to language barriers. All interviews and focus groups were audio-recorded, with two researchers present—one leading the discussion and the other taking field notes.

### Data Analysis

Data analysis was conducted iteratively, allowing interim findings to inform adaptations to the prototype intervention and interview guide. After data collection, we thematically analyzed the transcripts based on Braun and Clarke’s [27] six-phase framework. First, the four interviewers familiarized themselves with the data through listening to the recordings and reading the transcripts. Second, each interview was categorized and double-coded by JEL and RA using MAXQDA software (version 24; VERBI Software) and discussed with ARE and the broader team to discuss initial findings and identify knowledge gaps. Data from the focus groups were directly discussed within the team. In case of uncertainty, the most experienced qualitative researcher (EPMC) acted as an arbitrator. Third, key ideas and concepts were extracted to generate themes and subthemes. Fourth, these themes were collaboratively reviewed for consistency in interpretation. Fifth, the core research team (JEL, RA, RR, and ARE) refined theme names and definitions before developing a final narrative synthesis.

### Ethical Considerations

We received a waiver from the Medical Ethics Committee of the Amsterdam UMC (METC reference 2023.0578), as the study did not fall within the scope of the Medical Research involving Human Subjects Act (WMO Act) in the Netherlands. Informed consent was obtained from all participants before the start of each interview and focus group (Multimedia Appendix 4). Participants’ privacy was protected by deidentifying their data and replacing their names with confidential participant numbers. Participants were compensated for travel costs, where applicable. The intervention developed in this study will subsequently be evaluated for effectiveness and implementation in a randomized controlled trial (MIND PRO: ISRCTN registry: ISRCTN92928122) [28].

## Results

### Overview

Between October 2023 and November 2024, we interviewed 23 participants with a median age of 59 (IQR 55-63) years, of whom 65% (n=15) were female. The sample consisted of 14 participants with a Turkish migration background (10 female and 4 male participants; median age 55, IQR 50-59 y) and 9 participants with a South Asian Surinamese migration background (5 female and 4 male participants; median age 62, IQR 60-66 y). In the Turkish population, it was more challenging to include men. The focus group with Dutch individuals with low-SES was organized in an area housing mainly factory workers with generally low educational levels. This focus group consisted of 3 women and 10 men with a median age of 73 (IQR 68-73) years. A second focus group was organized at a community center with 7 Turkish women aged between 50 and 63 years to explore and refine contrasting perspectives observed in the individual interviews and to facilitate group discussion. As findings from the individual interviews with the South Asian Surinamese population were largely consistent, an additional focus group was deemed unnecessary. Our main findings are summarized in Multimedia Appendix 5, with supporting quotes from all three groups. Specific adaptations made to the mHealth intervention based on our findings are presented in Table 1. In describing the results, we focus on the overall blended mHealth intervention, which combines self-management of health factors with guidance from a remote coach. The app used by participants served as the delivery platform for the intervention.

**Table 1:** Adaptations made to the intervention based on findings.

Domain and goal				Adaptation in the intervention
**Usability and cultural adaptation of the intervention**				
	**Appropriateness**			
		Improve cultural and contextual fit	Provide the intervention in the first language of users Provide a culturally tailored intervention with advice and guidance that is sensitive to cultural preferences and needs	
		Enhance user acceptance	Provide adaptable font sizes Minimize the text in the app and use short videos as educational material Use a positive, motivating tone throughout the intervention	
**Key functionalities of the intervention**				
	**Goal setting**			
		Enhance self-efficacy	Users are motivated by the coaches to come up with their own goals	
		Achieve sustainable lifestyle changes	Coaches encourage participants to take small steps toward achieving their goals	
		Strike a balance between the flexibility to work toward several goals that are yet manageable	Participants can set a maximum of 2 goals at a time and are free to adjust their goals whenever they want	
		Coaches monitor whether goals are attainable to inhibit demoralization	The first goal will be set together with the coach using motivational interviewing techniques	
	**Self-tracking**			
		Sustain motivation	Personal performance feedback will be given by the coach Tracking should be nonobligatory without an abundance of reminders, as this may lead to demoralization and guilt toward oneself or the coach	
	**Educational material—general**			
		Improve trustworthiness	Provide videos with a familiar face, for example, their remote coach, to engage and instruct participants	
	**Educational material—diet**			
		Provide culturally sensitive information	Both culturally sensitive content that incorporates commonly used recipes and ingredients familiar in Turkish, South Asian Surinamese, and Dutch culture, as well as “general” healthy recipes	
	**Educational material—physical activity**			
		Provide culturally sensitive information	Content on physical activity provided options for indoor activities Being sensitive to the content about certain activities that are not common among Turkish people in the Netherlands (such as biking, which is very common in Dutch culture) Addition of dancing and household chores as physical activity goals	
	**Remote coach**			
		Build a bond of trust	After a first face-to-face meeting with the coach, users will be able to contact their coach via chat Participants will receive prerecorded videos of the coaches in the app to enhance familiarity	
		Provide culture-sensitive guidance	Coaches will be culturally matched and be able to speak Turkish if applicable	
**Implementation requirements**				
	**Autonomy**			
		Enhance the feeling of self-efficacy	Reduce notifications; too many messages are overwhelming Messages should be in a positive, nonobligatory tone; if not, they can be considered demotivating Coaches will match recommendations for physical activity to ability and preference to accommodate lifestyle changes at an individualized pace	
	**Social environment**			
		Engaging the social context	Participants are motivated to involve their social environment to participate in the lifestyle change	

### Usability and Cultural Adaptation of the Intervention

Participants were generally open to mHealth interventions and recognized the importance of a healthy lifestyle. However, most associated this primarily with overall well-being rather than the specific link between an unhealthy lifestyle and dementia risk in later life. User-friendliness of the platform was deemed essential in all three groups, and could be improved through minimal text, visual aids (photos or videos), adjustable font sizes, clear navigation between pages to prevent disorientation, and an intuitive design resembling familiar apps (Q3 and Q4 in Multimedia Appendix 5). While all participants owned a smartphone, many of them—especially those with a low SES—felt less competent with modern technologies compared to younger users, emphasizing the need for clear instructions on app use (Q4 in Multimedia Appendix 5). To improve the comprehensibility of the information in the app, participants emphasized the use of simple, easy-to-understand language. Specifically, participants with a Turkish background mentioned the importance of providing a Turkish translation. For them, it was not just about understanding the language but also about the emotional connection it created (Q1 in Multimedia Appendix 5). Many participants with a Turkish and South Asian Surinamese background emphasized the importance of cultural congruence (Q2 in Multimedia Appendix 5). They expressed a preference for culturally sensitive educational materials and a coach familiar with their cultural background, to better accommodate culture-specific needs and preferences.

### Key Functionalities of the Intervention

#### Goal Setting

Over half of the participants in all three groups wanted to work on a lifestyle goal. The most common goals were increasing physical activity, followed by improving dietary patterns and losing weight. The importance of personalization of goals was frequently mentioned, particularly by the focus group with participants with a low SES (Q7 in Multimedia Appendix 5). They also emphasized that a healthy lifestyle is not achieved by reaching a single health goal but requires working on multiple aspects, such as physical activity, healthy eating, and not smoking. Participants—mainly those with a Turkish background—highlighted that incorporating a healthy lifestyle into their daily routine can be challenging when it is not yet an established habit. Across all groups, participants reported feeling demotivated when goals seemed unattainable, and therefore preferred to take small, manageable steps toward lifestyle changes (Q5 and Q8 in Multimedia Appendix 5).

#### Self-Tracking

Most participants viewed the ability to track their own progress as motivating and a way to maintain accountability (Q9 in Multimedia Appendix 5). However, there was also some ambivalence toward self-tracking across all groups, as not reaching a goal might be demotivating (Q10 and Q11 in Multimedia Appendix 5). Using only positive reinforcement in the intervention was suggested as a solution to mitigate negative emotions associated with self-tracking. In addition, some participants mentioned the requirement to manually enter physical activity as a barrier. Comparing progress with others—whether on an individual level or a broader demographic level (ie, women of similar age and ethnic background)—was generally not perceived as desirable, particularly by women, as it could lead to feelings of jealousy, worthlessness, or guilt (Q12 in Multimedia Appendix 5).

#### Education Material

##### General

All participants were open to receiving information on a healthy lifestyle. They preferred practical information that is relevant to their personal health goals (Q15 in Multimedia Appendix 5). A key factor when seeking information was the perceived trustworthiness of the source (Q13 in Multimedia Appendix 5). Some expressed distrust toward digital resources, preferring to seek advice directly from their general practitioner (Q16 in Multimedia Appendix 5). In response to educational items presented in the prototype of the intervention, most participants indicated they would trust the information provided, particularly if it came from recognized experts or familiar figures, such as a lifestyle coach. Additionally, participants emphasized the importance of a positive, nonpressuring tone when presenting information (Q14 in Multimedia Appendix 5).

##### Healthy Eating

The need for cultural tailoring varied between the ethnic groups. South Asian Surinamese participants displayed strong adherence to their traditional dietary patterns (Q18 and Q19 in Multimedia Appendix 5), whereas the Turkish participants emphasized the significant diversity within Turkish dietary practices, even within Turkey itself (Q17 in Multimedia Appendix 5). This diversity facilitated their adaptation to new dietary habits, as they were already accustomed to cultural variation. In addition, integration into Dutch culture had already influenced their dietary patterns. In contrast, South Asian Surinamese interviewees stressed the historical significance and perceived health benefits of their traditional Hindustani cuisine, including Ayurvedic cooking (Q19 in Multimedia Appendix 5). Maintaining a healthy diet was often hampered by cultural factors, such as the social expectation to partake in abundant meals during gatherings. In both Turkish and South Asian Surinamese cultures, visits are traditionally accompanied by sweets, making dietary adjustments difficult. While some participants successfully modified their eating habits within their households, social settings—such as hosting visitors or dining with friends—posed greater challenges (Q20 in Multimedia Appendix 5). Specific advice on portion control (eg, “What is a healthy amount of rice?”) was suggested as a practical way to navigate these social influences.

##### Physical Activity

Several interviewees stressed the importance of finding the motivation for gradually increasing physical activity, as it is not inherently part of the Turkish or South Asian Surinamese culture (Q21 and Q24 in Multimedia Appendix 5). Turkish interviewees often mentioned the inability to cycle as a limitation (Q21 in Multimedia Appendix 5). A South Asian Surinamese woman highlighted her inability to swim, which limited her advised exercise options. The Dutch rainy weather was widely perceived as a significant barrier to engaging in outdoor physical activities, particularly among South Asian Surinamese participants. Some Turkish and South Asian Surinamese women favored home-based exercises as a more accessible alternative, with this preference being especially pronounced among Turkish women (Q22 and Q23 in Multimedia Appendix 5).

#### Remote Coach

Some participants from all groups were skeptical about combining a lifestyle app with a human coach (Q26 in Multimedia Appendix 5). This skepticism stemmed from a lack of experience with coach-supported interventions and concerns about intrusiveness, fearing it might undermine their autonomy in lifestyle decisions.

Others recognized potential benefits, viewing the coach as a source of information (especially on healthy food choices) and as motivation and accountability support (Q25, Q29, and Q30 in Multimedia Appendix 5). Most preferred contact on their own request via chat, voice messages, or video calls, with frequency ranging from daily to monthly. While many were initially hesitant, trust was a key factor in accepting a coach (Q26 in Multimedia Appendix 5). Participants, especially with a low SES, emphasized the need for a personalized approach, with tailored advice based on their individual situation (Q30 in Multimedia Appendix 5). Some also stressed the importance of building a relationship before feeling comfortable enough to ask questions (Q31 in Multimedia Appendix 5).

Preferences regarding cultural and gender matching varied. South Asian Surinamese participants did not consider cultural matching essential but emphasized that the coach should be knowledgeable about their dietary habits. Turkish participants, however, valued a Turkish-speaking coach for guidance. Some women from both ethnic groups preferred a female coach, believing they would better understand their concerns or help avoid tensions with their spouses (Q27 and Q28 in Multimedia Appendix 5).

### Implementation Requirements

#### Autonomy

Almost all participants across all groups explicitly mentioned that respecting their autonomy was essential for the intervention to be accepted as a tool for lifestyle change and to prevent disengagement. Most expressed an appreciation for receiving information, but stressed that guidance from the coach should be offered in a nonobligatory way and with positive messaging. The importance of respecting an individual’s autonomy was ubiquitous across all groups, and participants viewed this as an essential condition to be willing to participate in a blended intervention (Q32-Q34 in Multimedia Appendix 5).

#### Social Environment

Participants across all groups emphasized that their interpersonal relationships significantly influenced their ability to change their lifestyle. A supportive social environment that aligned with their personal health goals was seen as beneficial, such as having a partner join in the lifestyle change or friends supporting dietary adjustments (Q37 and Q40 in Multimedia Appendix 5). Conversely, many Turkish women and South Asian Surinamese men and women mentioned that family responsibilities often conflicted with their ability to change their lifestyle, reducing the perceived usefulness of the intervention (Q38 in Multimedia Appendix 5). Sharing progress with their social environment was seen as motivating by most participants in all three groups (Q36 and Q39 in Multimedia Appendix 5). However, it was stressed that this feature should remain optional. Not all participants felt the same about sharing progress; some regarded lifestyle changes as a personal journey, making comparisons difficult, while others feared that seeing others achieve greater success might discourage them.

## Discussion

### Principal Findings

This study explored the views and preferences of Turkish and South Asian Surinamese individuals, as well as Dutch individuals with low SES, regarding a blended mHealth intervention. Using the MIND-PRO app as a prototype, we assessed its usability and acceptability across these populations.

Participants recognized the importance of a healthy lifestyle for disease prevention but found it challenging to sustain long-term behavior changes. They indicated that the MIND-PRO intervention could assist them in making lifestyle changes and serve as a personalized guide with tailored information to keep them motivated and engaged. In the context of individuals with low SES and migration background who often have a shorter educational attainment, participants appreciated the app’s simple and familiar interface, which made it easy to navigate. Most participants identified at least one lifestyle goal they wished to achieve, highlighting the importance of making gradual, manageable changes. They regarded the app as a reliable source of information for healthy lifestyle education. Although tracking progress was seen as useful, some participants felt uncomfortable comparing their progress with others. To build trust, a face-to-face meeting with the coach and personalized advice were suggested, as remote coaching was unfamiliar to many. The ability to make independent choices within the intervention was particularly important, as participants valued autonomy in their decision-making. Social support played a significant role in behavior change, with encouragement from partners and peers viewed as beneficial, whereas family obligations were sometimes seen as a barrier. The expressed need for cultural tailoring varied. While some valued adaptation in dietary and physical activity guidance, Turkish participants felt their cultural diversity already facilitated adaptability to Dutch customs. In contrast, South Asian Surinamese participants remained more attached to traditional dietary patterns as part of their cultural identity. Across groups, there was a preference for culturally aware coaching, tailored language options, and personalized physical activity guidance.

mHealth interventions have demonstrated their potential in lifestyle modification for dementia prevention [29], and can be suitable for ethnically diverse populations if barriers related to accessibility and usability are addressed [12]. Nevertheless, most studies on mHealth interventions for lifestyle modification and healthy aging show only modest effects and have high attrition rates [13-15,20,29]. Strengthening engagement strategies is essential to use the full potential of mHealth for lifestyle modification.

Self-Determination Theory suggests that behavior change is more sustainable when it is driven by intrinsic motivation rather than external pressure [30]. This principle of choice and self-governance is incorporated into our intervention, allowing users to customize the intervention by setting personal goals and self-tracking their progress to enhance the feeling of autonomy. A remote coach can further support motivation by using techniques such as motivational interviewing to identify goals aligned with each user’s needs and values [31]. Positive reinforcement from a coach can improve usability, adherence, and health outcomes [32]. Furthermore, self-tracking is a well-established strategy to improve adherence, which this intervention supports through simple graphs and color-coded feedback in progress journals [33].

In addition to coaching, social interaction with peers is often mentioned as a valuable tool for behavior change, such as by enabling users to share their progress [34]. The study participants had mixed views on this: while some valued privacy and felt discouraged by others’ success, others found social support and a sense of healthy competition motivating. This contrast is supported by a systematic review on social features in mHealth interventions, which reported varying preferences for social involvement [35].

Culturally tailoring the intervention was deemed important by some participants, and previous studies have shown that tailored web-based interventions enhance patient engagement and are more effective at facilitating lasting behavior change [15]. However, cultural tailoring assumes that ethnic groups are homogeneous and share similar core values, whereas individuals bring their own unique social, cultural, and historical experiences. Both groups expressed the need for tailored physical activity guidance suited to individual abilities and skills. Facilitators for physical activity included exercising with peers and receiving suggestions for activities that could be done at home. To address these cultural nuances, the intervention will feature culturally sensitive content on topics such as language, diet, physical activity, and health goals, shared on users’ timelines to support lifestyle education (Figure 2). Such tailored content, aligned with cultural norms, beliefs, and practices, is easier to integrate into daily life [36], increases trust in the intervention, and improves users’ self-efficacy [15]. We aimed to balance cultural tailoring with personalization through features like goal setting, self-tracking, and personalized coaching. This dual approach acknowledges multiple cultural identities while remaining sensitive to individuals’ unique needs [37].

### Strengths and Limitations

A key strength of this study is its focus on diverse populations, particularly those from culturally diverse backgrounds or low SES, who are often underrepresented in preventive health care. Many mHealth interventions primarily target younger, highly educated users, potentially excluding individuals with lower health literacy, lower socioeconomic backgrounds, migration backgrounds, or older adults, thereby exacerbating health inequalities [38-40]. This highlights the need for inclusive interventions tailored to these groups [40]. Another strength is the use of a functional prototype, enabling iterative refinements based on direct participant feedback. Finally, the study benefited from a diverse research team with backgrounds in medicine, anthropology, mHealth, and public health, including members with Turkish, South Asian Surinamese, and Dutch backgrounds. This diversity enhanced cultural sensitivity and facilitated theoretical triangulation.

The study also has limitations. First, although a bilingual researcher (ES) facilitated communication with Turkish-speaking participants, the use of interpreters and translation may have led to a subtle loss of nuance or introduced bias, particularly given the cross-cultural nature of the discussions. However, as ES was part of the study team, she was able to indicate during the Turkish-language interviews which information was relevant and aligned with the aims of data collection. In addition, we engaged in regular reflection with her on culturally specific concepts that were challenging to translate. Second, the gender imbalance—characterized by underrepresentation of men among Turkish participants and overrepresentation of men in the focus groups with Dutch individuals with a low SES—limited our ability to assess gender-specific preferences. However, a similar prototype was previously tested extensively among Dutch individuals with low SES, where no significant gender differences in user preference and needs were observed [20]. Third, this intervention was tailored to the culturally specific preferences of Turkish and South Asian Surinamese populations in the Netherlands, as well as to the Dutch host population with a low SES. The transferability of our findings regarding culturally sensitive content to other contexts or populations is, therefore, limited. Future studies developing mHealth lifestyle interventions in other countries or for different target groups should explore and integrate the cultural needs of those populations. This study could offer a useful guide to which components may require adaptation, such as language, culturally sensitive educational content (eg, on physical activity and nutrition), and culturally aware coaching. At the same time, several of the core functionalities we identified—such as goal setting, self-tracking, accessible educational materials, and remote coaching—may be broadly applicable across diverse settings.

### Conclusions

This study highlights the importance of tailoring mHealth interventions for lifestyle modification to reduce dementia risk factors in migrants and individuals with low SES. To be effective, interventions should integrate cultural preferences, provide positive and nonobligatory content, and respect users’ autonomy while ensuring that information is relatable and easy to understand. A crucial factor for success is the active involvement of end users in the development process, allowing for the identification and integration of context-specific needs and preferences. Notable cultural differences emerged: South Asian Surinamese participants placed strong value on traditional dietary patterns, whereas Turkish participants prioritized language accessibility, preferring an app that ranged from general guidance to culturally tailored support. These findings underscore the need for a balance between cultural adaptation and personalization, ensuring inclusivity while addressing individual variation in preferences and needs.

## Appendix

Multimedia Appendix 1 COREQ checklist.

Multimedia Appendix 2 Topic list interviews.

Multimedia Appendix 3 Topic list focus groups.

Multimedia Appendix 4 Patient Informed Consent Form.

Multimedia Appendix 5 Summary of findings with supporting quotes.

## Abbreviations

COREQ: Consolidated Criteria for Reporting Qualitative research
HATICE: Healthy Aging through Internet Counseling of the Elderly
mHealth: mobile health
PRODEMOS: Prevention of Dementia by Mobile Phone Applications
SES: socioeconomic status
