# Supplementary material for: A Coach-Supported mHealth Lifestyle Intervention to Reduce Dementia Risk in Persons With Low Socioeconomic Status or a Migration Background: Qualitative Co-Design Study
Source: J Particip Med. 2025 Nov 4;17:e76094. doi: 10.2196/76094 (PMC12627971; doi:10.2196/76094)
Supplement: Multimedia Appendix 2 [file jopm_v17i1e76094_app2.docx]

# Interview Guide – the MIND-PRO app

## Introduction

## My name is [name], and I am a researcher at the Amsterdam UMC. These are [other people present during the interview]. I am part of a research group studying brain health.

## Together with you, we would like to explore how we can best design and improve the MIND-PRO app so that people are more likely to use it regularly and enjoyably. It greatly helps us to understand your needs and wishes.

## Since I will be asking for your opinions and experiences during this interview, everything can be said—nothing is strange or wrong. Please share as much as possible; we find all of it important and interesting.

## I would already like to thank you very much for taking the time to participate in this study.

## To ensure the interview runs smoothly, I’d first like to go over a few practical matters with you:

## We would like to record this interview. This makes it easier to listen back and process your responses later. Your anonymity is guaranteed, and everything you say will remain confidential. Once the study is completed, the recordings will be destroyed. If you are not comfortable with this conversation being recorded, please let me know. In that case, I will not record it and will only take notes during the interview.

## During our conversation, I will take notes for my own reference—this has nothing to do with the answers you give. I will place a large sheet of paper between us so that you can see what I write down.

## Our conversation will last about an hour. If you wish to stop or take a break at any point, please let me know.

## If you agree, I would like to begin and start the recording.

## Do you have any questions before we start the recording and begin the interview?

## --------------------------------------------- [start recording] ------------------------------------------------------

## Introduction – 5 minutes

## Before we begin, I’m curious to know who I’m speaking with.

## Could you first tell me a bit about yourself? [For example: partner, children, grandchildren, family, friends, distance to family and friends, work status, previous occupation, hobbies, daily activities.]

## Why did you decide to participate in this study? What would you like to gain from it yourself?

## Experience with smartphone use – 5 minutes

## Do you have a smartphone yourself? [or tablet, if only in possession of a tablet] o What do you mainly use it for? When do you use it? [only at home, or also on the go, e.g. in line at the supermarket?]

## Have you ever used an app to improve your lifestyle, such as apps for exercising more, eating healthier, etc.? o If the participant has used a health app before: Why (or why not)? What kind of app was it? When did you use it? How did that go? How did the app help you? How long did you use it? What made you use it less? o If the participant has never used a health app: Why not? What has prevented you from doing so? What held you back? What would you need to start using one?

## Introduction to the MIND-PRO app – 5 minutes

## Explain that this app was developed by a company and that we are now in the process of adapting it. Your opinion on the app is therefore highly valued, as there is still plenty of room for improvement.

## The researcher gives a short introduction about the app – (It is an app that can help you work on your lifestyle, set health goals, and track your progress.)

## The participant is then given the phone to explore the app at their own pace and share their first impressions.

## What is the participant’s first impression? How does it look? What feeling does it give you? Would you want to use it? What do you find appealing or less appealing?

## Does this app resemble any apps you currently have on your phone or tablet? What is different about it?

## Discussing personal health goals

## Explain that with the help of the app, a participant can work on certain lifestyle-related risk factors with the aim of keeping the brain healthy.

## Ask which lifestyle-related risk factors the participant has or would like to work on. This will serve as the starting point for the conversation and allows us to explore different parts of the app based on the participant’s needs and preferences.

## Views on (long-term) lifestyle change through a health app – 35 minutes

## As mentioned earlier, we want to improve the app. You now have a first impression of it, and I’d like to discuss its various components with you.

## For each topic (COACH, GOALS, GENERAL, PROGRESS, EDUCATIONAL MATERIAL), the interviewer gives a short introduction, followed by questions to the participant.

## COACH

## There is someone in the app who can help you with lifestyle change. What would you expect from such a person? How could they best help you? What would you call this person—buddy, coach, lifestyle expert...?

## What is the role of this person in your opinion? Motivation? Providing information? Confirmation? Advice? Help with using the app?

## Would it add value if the coach is culturally matched? Why yes/no?

## In the app, you can contact your coach/buddy/lifestyle expert [show chat page]. Is it clear how to reach out?

## Do you think you would want additional communication options?

## How often would you like to have contact with this person? Via chat or video call?

## GOALS

## How many health goals would you like to work on in the app? [show goal-setting questionnaire]. Would you work on multiple goals at once?

## GENERAL

## Do you think this app could help people in your surroundings? Why or why not? Could your family use this app?

## Could this app help you? Think, for example, of the risk factor we discussed earlier. Why or why not? What should such an app be able to do? What must it include for you to use it? [think of need for information, tracking weight, informational videos]

## What would initially motivate you to use such an app? o Follow-up: Would you do it to improve your quality of life or to prevent dementia?

## Do you think it would take much effort to use the app? Why or why not? What could make using it easier?

## What would you use the app for most? [Reading info? Setting goals? Contacting the coach? Tracking progress?]

## When do you think you would use the app? Once a week? Daily? At a fixed time? During the day or evening?

## What benefits do you expect from using the app? And what disadvantages?

## How do you think your surroundings would react if you used this app? Would they influence your use of it?

## Would you like to work on your health together with someone? If yes, would this be someone from your own circle or someone new?

## In what way would you prefer to work on your health together with someone? Why would that appeal to you? Would it affect your motivation? Would it make you more likely to use the app longer or more consistently?

## PROGRESS

## How would you like to measure your progress in the app? [show progress page in the app] How often would you track your progress? What do you think of these line graphs and this diary feature?

## Would you like to see how your progress compares to that of others?

## EDUCATIONAL MATERIAL

## What makes you trust or distrust certain information?

## Can you give an example of lifestyle information you’ve received before? What did you like or dislike about it? Did it inspire you to make changes? Why or why not?

## Who do you currently listen to or take information from? Doctor? Family? Friends? Why do you (or don’t you) listen to them?

## Would you like to hear personal experience stories?

## How often may we send you push notifications? What kind of information would you find relevant to receive this way?

## Closing the interview – 5 minutes

## We’ve come to the end of the interview. I really appreciate your stories and experiences, and I hope you’ve felt comfortable being yourself during the interview.

## Is there anything else you’d like to add? Do you have any questions for me?

## Thank you very much for your cooperation and openness. If you have any questions or comments, please feel free to contact us.
