# Supplementary material for: A Coach-Supported mHealth Lifestyle Intervention to Reduce Dementia Risk in Persons With Low Socioeconomic Status or a Migration Background: Qualitative Co-Design Study
Source: J Particip Med. 2025 Nov 4;17:e76094. doi: 10.2196/76094 (PMC12627971; doi:10.2196/76094)
Supplement: Multimedia Appendix 3 [file jopm_v17i1e76094_app3.docx]

# Focus Group Guide – Brain Health with an App

For those who are participating in this study for the first time: welcome! For those we have already spoken to in individual interviews: thank you once again for your participation!

We are [names of all attending researchers, moderators, observers, and mHealth developers → each attendee briefly explains why they are present and what their role will be].
We are part of a research group studying brain health, particularly the prevention of dementia.

An app has been developed for smartphones and tablets that can be used to work on a healthier lifestyle together with a coach. Together with you, we want to explore how we can best design this app to increase the likelihood that people will actually use it—and enjoy doing so. It helps us tremendously to understand your needs and preferences.

During this workshop, we will explore the app together and ask for your opinions about it. We kindly ask you to respect each other’s viewpoints, but also not to be afraid to respectfully disagree.

We would like to sincerely thank you in advance for taking the time to participate in this study.

Before we start, I would like to go over a few practical matters with you:

- We would like to record this workshop. This will make it easier to listen back and process your responses later. Your anonymity is guaranteed, and everything you say will remain confidential. Once the research is completed, the recordings will be destroyed.
- During our discussion, notes will be taken to help remember key points. This has nothing to do with the content of your answers.
- The workshop will last about one hour. If you wish to stop or take a break at any time, please feel free to do so.

Has everyone understood this, and are there any questions?
Do you all agree that I start the recording now?

Do you have any questions for me before we start the recording and begin the session?

--------------------------------------------- **[start recording]** ------------------------------------------------------

Depending on the size of the group, we will divide participants into smaller subgroups of 3–4 people to think through the different tasks together. Each group will receive one phone with the app installed.

**1. Task 1 – Introduction / Ice-breaker – 10 minutes**

**Start:** Brief explanation of the app – setting your own goals and having a coach.

First, we would like to ask you to explore the app and set a goal within it. Please do this together in your group, and then let us know how it went.

We will then take turns letting each group share their first impressions of the app, making sure that everyone who wants to speak gets the opportunity to do so.

**2. Task 2 – Setting goals and tracking progress – 20 minutes**

(These two questions will be discussed separately in subgroups, followed by collective feedback.)

**QUESTION 1:**
Now that you have set a health goal in the app, we’d like to hear your opinions on a few things:

- Imagine you are working on a goal—would you adjust your goal along the way?
- Why or why not? If yes, how often would you do this?

**QUESTION 2:**
Imagine you are working on a goal in the app:

- Would you track your progress in the app? Please take a look at the progress page. What do you think of it?
- If you track your progress, would you like to compare it? Compare your progress to 1) people similar to you, or 2) the guideline.
- Would tracking your progress motivate you? Why or why not? Would comparing motivate you? Why or why not?
- Would you like to share your progress with family and friends?

**3. Task 3 – Content, frequency, and contact with coach – 20 minutes**

(These two questions will be discussed separately in subgroups, followed by feedback.)

We have now discussed some practical matters, and we would like to talk with you about the app’s content.

**QUESTION 1:**
*Cultural sensitivity* – While using the app, you will receive a lot of information. We would like to know what kind of content you would enjoy or find interesting to read. What kind of information fits your daily life? What kind of information connects to your culture? For example: healthy recipes, tips for staying active, etc.

**QUESTION 2:**
*Coach* – There is someone in the app who can help you with lifestyle changes and goal setting. What would you expect from such a person? How could they best support you? How often would you like to speak with this person? Should the contact be initiated by you or by the coach? Only when you have a question, or regularly?

**Additional question:**
What would help you most in setting and achieving a lifestyle goal?
Four options:

1. The app with a coach
2. The app alone
3. Only an online coach
4. On your own

During Tasks 2 and 3, the mHealth developers will make live adjustments to the app. Then, in Task 4, we will test these changes with the participants.

**4. Task 4 – Testing the app together – 15 minutes**

Throughout this workshop, we have shaped the app together and made adjustments based on your suggestions. Now, we’d like to go through the app again with you and test the changes or ideas you’ve provided.

Afterward, participants will again have the opportunity to share their opinions on the modifications made to the app.

**5. Closing the workshop**

We have come to the end of the workshop. We greatly appreciate your participation and your suggestions—thank you so much for your valuable input!

- Is there anything else you would like to share? Do you have any questions for us?

Thank you very much for your cooperation and openness. If you have any questions or comments, please feel free to contact us.
