# Supplementary material for: A Coach-Supported mHealth Lifestyle Intervention to Reduce Dementia Risk in Persons With Low Socioeconomic Status or a Migration Background: Qualitative Co-Design Study
Source: J Particip Med. 2025 Nov 4;17:e76094. doi: 10.2196/76094 (PMC12627971; doi:10.2196/76094)
Supplement: Multimedia Appendix 4 [file jopm_v17i1e76094_app4.doc]

### patient informed consent form

**Disclaimer: This is a not a certified translated version of the original Dutch & Turkish Patient Information form and Informed Consent form.**

# Information for Participation in the MIND-PRO Study


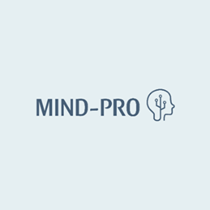

Dear Reader,

With this information letter, we would like to ask for your opinion on an app. Participation is voluntary.
We are a group of researchers who have developed an app to help people lead healthier lives. We created this app because we know that living a healthier life can potentially reduce the risk of dementia. We would like to know your thoughts on this app. Your feedback can help us improve it.

This document explains what participation entails and what is expected of you if you decide to take part.

 Are you interested?
 - Please read this letter carefully.
 - Ask questions to the researcher who provided this information.
 - If you wish to participate, fill out the attached form.

## General Information

The research is conducted by a research group at Amsterdam UMC.

## What is the Purpose of the Research?

The goal of this research is to improve an existing app. This app was developed to help people live healthier lives, thereby potentially reducing the risk of dementia. Research shows that dementia has multiple causes, some of which are associated with an unhealthy lifestyle. Examples include smoking, obesity, insufficient physical activity, and high blood pressure. Living a healthier life can be challenging. That’s why we developed an app to assist with this. We would like to ask for your feedback on the app. We aim to collaborate with you to make the app as good as possible. Your input is very valuable to us.

## What Happens if You Participate in the Study?

If you decide to participate in this study, you may be invited to various components of the research. You can choose which parts you want to participate in.

**Part 1**: You will first be invited for a conversation lasting about 45 minutes. This can take place at your home or at Amsterdam UMC. Audio recordings will be made during the conversation, but only with your permission.

**Part 2**: You will then be invited to a group discussion lasting approximately 60 minutes. During this session, we will review and use the app together. We will ask for your honest feedback. This session will likely take place at Amsterdam UMC, and audio recordings will also be made here.
During the study, we will ask for your date of birth and information about your health and lifestyle. If you agree, we may also ask about your ethnicity.

## What Does Participation Mean for You?

You will contribute to the development of our app for further research. Keep in mind that participation requires your time.

## What if You Don’t Want to Participate or Wish to Withdraw?

Participation is entirely voluntary. You can decide to stop at any time during the study without providing a reason. However, you should notify the researcher immediately. Data collected up to that point will still be used for the study.

## How Do We Handle Your Data?

If you participate, you consent to the collection, use, and storage of your research data for the purposes of this study.

Why do we collect, use, and store your data?

We do this to answer the research questions of this study.

What happens to audio recordings?
Audio recordings may identify you. These recordings will be transcribed, and the audio files will be destroyed afterward.

How do we protect your privacy?

To protect your privacy, your data will be coded. Only the code will be used, and identifying information will not be shared. The key to the code will be securely stored at Amsterdam UMC. Only the research team will have access to it. Reports and publications will not include any information that can identify you.

How long do we keep your data?
Your data will be stored for 10 years at the University Medical Centre after the study ends.

Can you withdraw consent for your data?
You can withdraw your consent at any time, and your data will be deleted.

Can we contact you for follow-up research?
 If there is a follow-up study, we may contact you to ask if you’d like to participate. You can indicate this on the consent form.

## Will You Be Compensated?

Participants will receive a GVB day pass to cover travel costs. Lunch will also be provided during the group discussion.

## Do You Have Questions?

This study has been reviewed by the non-WMO review committee at Amsterdam UMC. For questions, contact Anne Roos van der Endt at mind-pro@amsterdamumc.nl.

## Do You Have a Complaint?

If you have a complaint, you can discuss it with the researcher or contact patient support services.

Consent Form

- I have read the information letter. I was able to ask questions, and my questions have been answered sufficiently. I had enough time to decide whether to participate.
- I understand that participation is voluntary and that I can decide to stop participating at any time without giving a reason.
- I consent to the collection and use of my data as described in the information letter.
- I consent to my data being stored for 10 years after this study within Amsterdam UMC, AMC location. This storage period of 10 years is required by law.

- I agree to participate in this study.

Please check yes or no below:

- I consent to the collection and use of audio recordings. These recordings will be destroyed after transcription.
 O Yes O No
- I consent to being contacted for follow-up research after this study.
 O Yes O No

Participant's Name:
Signature: Date: __ / __ / __

-------------------------------------------------------------

I declare that I have fully informed this participant about the mentioned study.
If any information arises during the study that may influence the participant's consent, I will inform them in a timely manner.

Researcher's Name:
Signature: Date: __ / __ / __

-------------------------------------------------------------

The participant receives a complete information letter along with a copy of the signed consent form.

### Interview guide

**Disclaimer: This is a not a certified translated version of the original Dutch & Turkish Interview guide**

## Introduction

I am [name], a researcher at the Amsterdam UMC, and these are [other attendees during the interview]. I am part of a research group studying brain health.

A company has developed an app that we would like to improve. This app helps users work on healthy behavior with the support of a coach.

Together with you, we want to explore how best to design and improve this app to increase the likelihood of people enjoying and actively using it. Understanding your needs and preferences helps us significantly. Ultimately, the goal is to enable users to manage their health independently, with the coach available for assistance.

Since I’ll be asking for YOUR opinion and experience during this interview, there are no wrong answers, and you can share anything. The more you share, the better—we value all your input.

Thank you in advance for taking the time to participate in this research.

To ensure the interview goes smoothly, I’d like to review some practical matters:

- We would like to record this interview to make it easier to review and process your responses later. Your anonymity is guaranteed, and everything you say will remain confidential. Once the research is complete, the recordings will be destroyed. If you’re uncomfortable with the recording, please let me know; I will then refrain from recording and only take notes.
- I will take notes during our conversation to help me remember details. This has no impact on your answers. I will use a large sheet of paper for note-taking, placed between us so you can see what I’m writing.
- The conversation will last approximately 30 to 45 minutes. If you wish to stop or pause at any time, feel free to let me know.

If you agree, I would like to begin and start the recording.

Do you have any questions before we start?

## Informed Consent

- Review the informed consent form with the participant.
- Both the researcher and participant sign the form.

## Getting Acquainted

Before we dive in, I’d like to learn a bit more about you.

Could you tell me about yourself? (e.g., partner, children, grandchildren, family, friends, distance from family and friends, work status, former profession, hobbies, daily activities.)

Why did you decide to participate in this research? What do you hope to gain from it?

## Experience Using a Smartphone

- Do you own a smartphone? (Or a tablet, if you only own a tablet.)
- What do you mostly use it for? When do you use it? (e.g., at home on the couch or while on the go, in line at the grocery store.)
- Since when have you had a smartphone/tablet? Do you find it easy to use? If not, do you receive help with using it?

#### Experience with Lifestyle Apps

- Have you ever used an app to improve your lifestyle (e.g., apps for exercising more, eating healthier)?
  - If yes: Why or why not? What kind of app was it? When and how did you use it? How did the app help you? For how long did you use it? What caused you to use it less or stop using it?
  - If no: Why haven’t you used a lifestyle app? What prevented or discouraged you? What would help you to start using one?

## Introduction to the MIND-PRO-App prototype

Explain that this app was developed by a company and is currently being adapted. We value your opinion because there’s still plenty of room for improvement.

- Briefly introduce the app: “It is an app designed to help you work on your lifestyle goals and track your progress.”
- Let the participant explore the app on a smartphone or tablet and share their initial impressions:
  - What’s your first impression of the app?
  - How does it look?
  - What feelings does it evoke?
  - Would you want to use it?
  - What do you like or dislike about it?

## Discussing Personal Health Goals

Explain that the app allows participants to address certain lifestyle-related risk factors aimed at lowering dementia risk.

Ask about any lifestyle-related risk factors they have or want to work on. Use this as a starting point to discuss different features of the app.

## Vision on Long-Term Lifestyle Changes via a Health App

Discuss different aspects of the app, such as the COACH, GOALS, GENERAL FEATURES, SELF-TRACKING, and EDUCATIONAL MATERIALS.

**COACH**

- Explain the coach's role: A person who helps set goals supports via the app.
- Questions:
  - What do you expect from such a person?
  - What would you call them (e.g., buddy, coach, lifestyle expert)?
  - What role should they play (e.g., motivator, information provider, advisor)?
  - Would you find videos from a coach helpful?
  - Do you think it is important that the coach has the same cultural background?

**GOALS**

- Wat do you think of the goal-setting feature?
- How many health goals would you like to work on simultaneously?

**GENERAL**

- Do you think this app could help you or people in your environment?
- What would motivate you to use such an app?
- What features should it have to make it user-friendly?

**SELF-TRACKING**

- How would you like to track your progress?
- How can it help in reaching your goals?

**EDUCATIONAL MATERIALS**

- What type of information do you trust?
- How do you like to receive information? And from whom?
- Would you like to hear stories from others?

## Wrapping Up

We are nearing the end of the interview.

- Do you have any final thoughts or questions?
- Would you be interested in participating in a group discussion?

Thank you very much for your openness and time. Feel free to reach out if you have any additional questions.

### Guide Focus Groups

**Disclaimer: This is a not a certified translated version of the original Dutch & Turkish Focus group guide**

## Introduction

Welcome everyone, thank you for your participation in this study.

We are [names of all present researchers, moderators, observers, and mHealth developers -> each attendee will briefly explain why they are present and what their role will be], and we are part of a research group studying brain health, particularly the prevention of dementia. An app has been developed for smartphones or tablets, which can be used to self-manage healthy behavior with the help of a remote coach. We want to collaborate with you to figure out how you we optimize this app so that people would like using it. It helps us immensely to understand your needs and preferences regarding the use of this app.

During this workshop, we will examine the app together and ask for your opinion. We kindly ask you to respect each other's opinions but also not to be afraid to respectfully disagree.

To ensure a smooth workshop process, I would first like to go over a few practical matters with you:

- We would like to record this workshop. This makes it easier to review and process your responses afterward. Your anonymity is guaranteed, and everything you say will remain confidential. Once the study is complete, the recordings will be destroyed.
- During our discussion, notes will be taken to help us remember certain aspects; this has nothing to do with your answers.
- The workshop will last approximately 1 hour. If you wish to stop or take a break, you can indicate this at any time.

Does everyone understand this, and are there any questions? Do you agree that I will now start the recording?

Do you have any questions before we start the recording and begin the interview?

---------------------------------------------[Start recording]------------------------------------------------------

Depending on the group size, we will divide participants into smaller groups of 3-4 people who will reflect on the different tasks together. Each group will receive one phone with the app installed.

## Task 1 – Introduction/Ice-breaker

First, we ask you to explore the app and set a goal in the app. Do this together in your group and then let us know how it went.

Each group will take turns sharing their first impressions of the app. We will ensure that everyone who wishes to speak has the opportunity to do so.

*During Tasks 2 and 3, the mHealth developers will make live adjustments to the app, which will then be tested with the participants in Task 4.*

## Task 2 – Setting Goals and Tracking Progress

(Two questions to be discussed separately in subgroups, followed by feedback.)

**QUESTION 1:** Now that you have set a health goal in the app, we would like to hear your opinion on a few things:

- Imagine that you are working on a goal; would you adjust your goal along the way?
- Why would you, or why not? If so, how often would you do this?

**QUESTION 2:** Imagine you are working on a goal in the app:

- Would you track your progress in the app? Please explore the self-tracking page. What do you think of it?
- If you track your progress, would you like to compare your progress? Compare progress with 1) people similar to you or 2) a general guideline.
- Would tracking your progress be motivating? Why would it be, or why not? Would comparison be motivating? Why would it be, or why not?
- Would you like to share your progress with family and friends?

## Task 3 – Content Information and Frequency + Contact with Coach

Now that we have discussed some practical aspects, we would also like to discuss the app's content with you.

**QUESTION 1:** While using the app, you will receive a lot of information. We would like to know what kind of (cultural sensitive) content you would find interesting or enjoyable to read. What information aligns with your daily life? What information fits with your culture? For example, healthy recipes, tips for staying active..

**QUESTION 2:** Coach: There is someone in the app who can help you with lifestyle changes and goal setting. What would you expect from such a person? How could this person best help you? How often would you like to speak with this person? Should the contact be initiated by you or by the coach? Should it only happen when you have a question, or should it be regular?

**Extra Question:** What would help you the most in setting and achieving a lifestyle goal? Four options: the app with a coach, the app alone, an online coach only, or by yourself.

## Task 4 – Testing the App Together

During this workshop, we have collaboratively shaped the app and adjusted it based on your suggestions. Now, we would like to go through the app together once more to review and test the changes and tips you have provided.

Participants will then again have the opportunity to share their opinions on the adjustments made to the app.

## Workshop Conclusion

We are nearing the end of the workshop. We greatly appreciate your participation and suggestions—thank you very much!

- Is there anything else you would like to add? Do you have any questions for us?
- We may want to organize a similar session again in the future. May we invite you for that? Or do you know people who would like to participate in such a session?

Thank you very much for your cooperation and openness. If you have any further questions or comments, please feel free to contact us.
