# Supplementary material for: A Coach-Supported mHealth Lifestyle Intervention to Reduce Dementia Risk in Persons With Low Socioeconomic Status or a Migration Background: Qualitative Co-Design Study
Source: J Particip Med. 2025 Nov 4;17:e76094. doi: 10.2196/76094 (PMC12627971; doi:10.2196/76094)
Supplement: Multimedia Appendix 5 [file jopm_v17i1e76094_app5.docx]

| Findings uniform across all groups | Differing views between groups | Group | Quote |
| --- | --- | --- | --- |
| Usability and cultural adaptation of the intervention | | | |
| - Minimal text and easy to understand language is preferable. Make use of figures and videos. - The app should work intuitively and resemble other commonly used app (such as the chat and Whatsapp). - It should be easy to navigate between pages without small buttons. - Small text fonts should be adjustable - The older target population felt less tech-savy and appreciated good instructions in how to use the intervention | - The content off the app should be available in native language for the Turkish population. - Turkish and South Asian Surinamese individuals prefer a culturally tailored app. | Turkish background (T) | 1. *“Right, yes, yes, so it’s just a bit cold. You know, how can I explain it? AR: The Dutch? P: Yes, yes, so fake does it feel all of a sudden. I know, it’s not fake, and it not very weird or that I don’t trust it, but still it feels cold to me.”(female, 52 yr)* |
|  |  | South-Asian Surinamese (SAS) | 1. *“If there are studies on ethnicity for populations such and Hindustan and Turkish people, they can get specific help. Because now, everything - pharmaceuticals and everything - is focused on the white population. The question is, does it help us as well?” (male, 51 yr)* |
|  |  | Low-SES | 1. *"It's too crowded, the boxes are too small, and my fingers are too thick. The font size needs to be bigger. We had the problem that we pressed the wrong key, and then it disappeared, so we had to start all over again." (focus group)* 2. *“you see, not everyone here is used to working with a smartphone.” (focus group)* |
| Key functionalities of the intervention | | | |
| Goal setting | | | |
| - Individuals wanted to choose their own goals - Goal setting should be flexible and adaptable - Goals should be attainable. Taking small steps can help in achieving sustainable lifestyle changes | - Some participants mentioned the need to work on several goals at the same time, while others stressed the importance of taken small steps. Highlighting the importance of self-managing goals and personalization. | T | 1. *“Yes, and making it very accessible. […] I used to think that if I wanted to be active, I always had to walk or cycle for an hour with my husband. […] But I get tired so quickly—too tired—and it frustrates me. Then my friend gave me advice. I hadn't thought of it myself, and she said, "Why don’t you walk for just 20 minutes instead?" (female, 58 yr)* |
|  |  | SAS | 1. *“Preferably I want to work on all three goals simultaneously (physical activity, lowering cholesterol and weight loss). […] Because all three are very important to me.” (female, 66 yr)* |
|  |  | Low-SES | 1. *“Yes, we all have different goals, right? Its personal, maybe one person has high cholesterol, another is overweight, so you need to be able to make your own choices.” (focus group)* 2. *“If you try to work on too many goals at the same time, I can imagine you might feel like you're spending the entire day just noting things down and keeping track, and then it stops working. […] It shouldn't become too difficult. “ (focus group)* |
| Self-tracking | | | |
| - Self-tracking is perceived as helpful as long as its non-obligatory - Works best when it is visualized in simple graphs or diaries - Will be used to keep themselves accountable and sustain motivation - Most people did not feel the urge to compare their progress with peers or share their progress with others. Could lead to demotivation if they feel they are underperforming | - Generally women preferred to not compare progress to others, not even on a demographic level. | T | 1. *“I just want to see it, and if I achieve the line (goal), then I’m very happy. Maybe it will motivate you like, come on, you can move the line upwards by doing a little more.” (female, 55 yr)* 2. *“It has a good side and a bad side. For example, one might say, ’Wow, I’ve already taken 10.000 steps today’, but then you step on the scale and see you haven’t lost a kilo - that can demotivate you.” (male, 60 yr).* |
|  |  | SAS | 1. *"And then you get startled, like, well, I haven't exercised. What am I supposed to write? Should I lie or tell the truth?" (female, 59 yr)* |
|  |  | Low-SES | 1. *On comparing progress: “That’s a difficult question. Every person is different. The goal might be the same, but the way you work on it can be different. It is difficult to compare” (focus group)* |
| Education material – general | | | |
| - Practical and tailored information is perceived as helpful in achieving health goals - Trustworthiness is an important aspect. - Familiarity (knowing the coach) can add to this trustworthiness. - Information should be framed positively, which is perceived as more motivating. |  | T | 1. *“Yes, or I go to my GP, but not to the internet. Because, if you search online, “I have a headache, what’s wrong?” suddenly it tells you that you have a tumor in your head.” (female, 52 yr).* |
|  |  | SAS | 1. *“I am happy if it motivates me, that I am doing something positive and if it (the app) also brings me positivity.” (female, 59 yr)* |
|  |  | Low-SES | 1. *In the library, I can find a lot of practical information which is nice, like cooking with less oil of less fat, that is just practical.” (focus group)* 2. *“ I do think that someone who has really been trained for it (referring to their general practitioner) and knows all the aspects is better at giving advice.” (focus group)* |
| Education material - diet | | | |
|  | - Turkish participants expressed the willingness to adjust their diet. - South Asian Surinamese individuals saw diet part of identity, therefore rigid and difficult to change. - For both Turkish and South Asian Surinamese individuals social gatherings and holidays often accompanied with an abundance of food, rude to so “no”, therefore difficult to maintain a healthy diet at times. | T | 1. *“But it is half-half now. Half Dutch culture, half Turkish.[…] Eating a sandwich in the afternoon, whereas in Turkish cuisine, it is warm food.” (female, 62 yr)* |
|  |  | SAS | 1. *“It’s different you see, you eat potato’s we eat rice (in the Surinamese kitchen).” (male, 64 yr)* 2. *“South-Asian Surinamese people have been eating this since 5000 years B.C. And the Ayurvedic kitchen is rich.” (male, 58 yr)* 3. *“I can’t (ask other people to cook differently). Then she has to take so many things into account; whole grain rice, no salt, no sugar… No, that just won’t work.” (female, 66 yr)* |
|  |  | Low-SES |  |
| Educational material – physical activity | | | |
|  | - Barriers to go to the gym, especially in Turkish women. - For South Asian Surinamese participants bad weather is barrier for outdoor activities | T | 1. *“No, that’s not possible [cycling]. Maybe if a group of women starts walking slowly. They’ll start slowly and then it becomes more, more, more.“ (female, 58 yr)* 2. *“I wanted to do sports. […] I do it at home. (The gym) was too expensive so I quit. (female, 50 yr)* 3. *Many Turkish woman are at home a lot so (doing physical activity) at home during the day should be attainable. (female, 55 yr)* |
|  |  | SAS | 1. *“But also, I think in general, you need to motivate Hindustans to start exercising. […] It’s not part of the culture. It’s not ingrained.” (male, 58 yr)* |
|  |  | Low-SES |  |
| Remote coach | | | |
| - A face-to-face conversation is a requirement, allowing a relationship of trust to be built and enabling the coach to become aware of the personal situation. - Remote coaches should guide users in a non-obtrusive way to maintain a sense of autonomy. - Coaches are mainly viewed as a source of information and to keep users accountable. | - Turkish and South Asian Surinamese women prefer a female coach - South Asian Surinamese individuals did not perceive a culturally matched coach as requirement, but the coach should have knowledge on dietary habits. - Participants with low-SES viewed personal guidance as essential | T | 1. *“It (the coach) should motivate them (the app user), not just tell them what to do.” (male, 63 yr).* 2. *“Look like here with you we have this conversation. I don’t know how that happens with the coach, I don’t know him/her […] I should have trust first and then (I will ask questions). I always have that with people.” (female, 59 yr)* 3. *“Doesn’t matter (having a coach with Turkish or Dutch ethnic background). […] But yes, a woman, because she knows. […] We can share that experience. […] She’ll understand that feminine part.” (female, 59 yr)* |
|  |  | SAS | 1. *"Yeah, maybe because it feels familiar or something, like when I tell my husband, I'm going to talk to the coach. [...] And yeah, then my husband doesn't have to say, 'Are you talking to that man again?' You know?" (female, 62 yr)* 2. *“The more, the better (contact frequency with the coach). Because then you know that someone is watching over you. Like, you’re heading the wrong direction.” (female, 59 yr)* |
|  |  | Low-SES | 1. *“If it’s a personal coach who can keep track of your progress and has looked at your data, and there are things you can work on, then it can be useful to get advice.” (focus group)* 2. *“Chatting with the coach would be easier after a personal conversation as the coach will already know who you are. […] And if you don’t know who is sitting on the other side (of the chat) you will not ask questions.” (focus group)* |
| Implementation requirements | | | |
| Autonomy | | | |
| - Reduce notification, too many messages is overwhelming - Messages should be in a positive, non-obligatory tone, they are otherwise demotivating - Flexibility in the app is important, everyone changes their lifestyle at a different tempo, there should be room in the app for this |  | T | 1. *“Every time the phone paged me, it got on my nerves within just two days […] Yes, the following week, I went to my doctor and said: ’I’m going to remove this app.” (female, 54 years old)* 2. *“The app would feel restrictive.. I can’t stand it when someone tells me what I should or shouldn’t do or eat.” (male, 49 yr)* |
|  |  | SAS | 1. *“ I don’t like to receive such messages (push messages), then you’re being pushed. And I don’t want that. I don’t like it if someone pushes me to do something. Not at work and from such a machine (telephone with app). (male, 62 yr)* |
|  |  | Low-SES |  |
| Social environment | | | |
| - Being physically active together is more motivating and improves sustainability - An understanding and motivating partner is a big facilitator | - Especially amongst Turkish and South Asian Surinamese individuals, children could play an important role in motivating their elders to change their lifestyle. | T | 1. *“I think together (preference for working on lifestyle), because alone it gets boring soon. And if you go with a group, then that motivates and by looking at others you can get challenged.” (male, 60 yr)* 2. *“I have a 17-year old daughter. She’s like a true coach. She says, ‘Mama, you eat too much candy, you eat way too sweet.” (woman, 50 yr).* |
|  |  | SAS | 1. *“So from the moment you are born, you are in service of another (your family). Hindustan parents take this very seriously. So your children are your everything and go before anything else, even before your own health.” (male, 58 yr)* |
|  |  | Low-SES | 1. *And of course, it’s nice when you train together, and you arrive in class and say. ‘ Hey, look how I have been working (on my goals).’ See, I made progress and then you can get a compliment, I’m really sensitive to that.” […] “ it’s not much about looking at the progress, because it’s individual. But you can you it like. ‘Hey, I struggled with this, and then you can help each other.” (focus group)* 2. *“ What’s also very important is that you share this with your partner or spouse.” (focus group)* |

Legend:

T = Individuals with a Turkish migration background

SAS = Individuals with a South-Asian Surinamese migration background

Low-SES = Individuals with a low socio-economic status with a native Dutch background
